# Resistive switching and its suppression in Pt/Nb:SrTiO_3_ junctions

**DOI:** 10.1038/ncomms4990

**Published:** 2014-06-02

**Authors:** Evgeny Mikheev, Brian D. Hoskins, Dmitri B. Strukov, Susanne Stemmer

**Affiliations:** 1 Materials Department, University of California, Santa Barbara, California 93106, USA; 2 Department of Electrical and Computer Engineering, University of California, Santa Barbara, California 93106, USA

## Abstract

Oxide-based resistive switching devices are promising candidates for new memory and computing technologies. Poor understanding of the defect-based mechanisms that give rise to resistive switching is a major impediment for engineering reliable and reproducible devices. Here we identify an unintentional interface layer as the origin of resistive switching in Pt/Nb:SrTiO_3_ junctions. We clarify the microscopic mechanisms by which the interface layer controls the resistive switching. We show that appropriate interface processing can eliminate this contribution. These findings are an important step towards engineering more reliable resistive switching devices.

## Abstract (web summary)

Understanding the resistive switching mechanism in oxide-based memories is an ongoing challenge. Here, the authors isolate an unintentional interfacial layer as the origin of resistive switching in Pt/Nb:SrTiO_3_ junctions, and show that suitable processing can remove this unwanted contribution.

## Graphical abstract

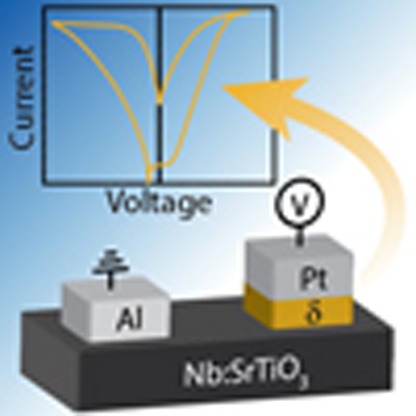

Non-volatile resistive switching devices have attracted considerable attention, owing to the promise of a non-volatile, continuously tunable (as opposed to binary) memory and new computing approaches^1^,^2^,^3^. The two-terminal nature of such devices allows for lateral scaling to nanometre dimensions^4^. Typical devices show, however, poor reproducibility and device-to-device variability, which are the main barriers towards creating a viable technology^3^,^5^. One extensively studied type of resistive switching device consists of a Schottky junction between a doped, wide-band gap oxide, such as SrTiO_3_, and high work-function metals such as Pt, Au or metallic oxides such as SrRuO_3_ and YBa_2_Cu_3_O_7−*x*_^6^,^7^,^8^,^9^. In such devices, resistive switching is accompanied by a modulation of the effective Schottky barrier height^7^,^8^,^10^,^11^,^12^. These devices combine technologically attractive features, such as hysteretic current–voltage (*I*–*V*) characteristics with large on/off ratios, bipolar switching and continuously tunable resistance states. Unlike other types of resistive switching memories, they do not require an initial forming step, which is a significant advantage for practical applications. To effectively address the issues of reproducibility and variability, the microscopic origins of resistive switching must be understood.

Here, we report a systematic study of resistive switching of metal/oxide interfaces formed between Pt and Nb-doped SrTiO_3_. The results demonstrate that resistive switching is controlled by an interfacial layer, as revealed by a parasitic interfacial capacitance, an increased ideality factor of the Schottky barrier and an extended depletion width within the SrTiO_3_. A charge trapping-based model can fully explain the Schottky barrier lowering that accompanies the resistive switching. The interfacial layer capacitance is crucial in controlling the magnitude of the effect. We show that the contribution of such unintentional layers to the resistive switching process can be minimized by appropriate processing, thus providing a pathway towards engineering more reliable resistive switching devices.

## Results

### *I*–*V* characteristics

**Figure 1:**
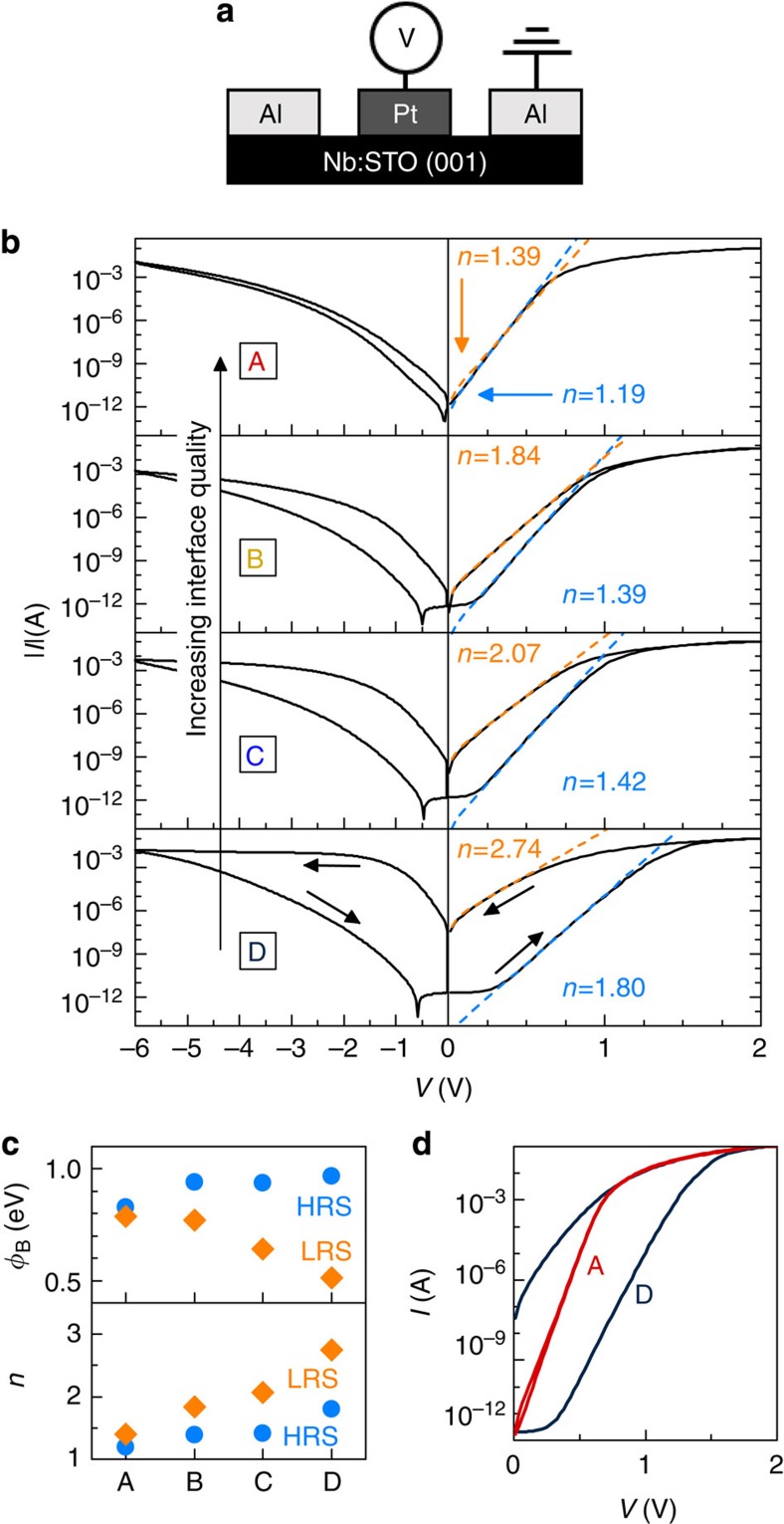
*I*–*V* characteristics of the devices. (**a**) Schematic of the device. (**b**) *I*–*V* characteristics for all samples. The blue and orange lines are fits to equation (1) for the HRS and the LRS, respectively, in each case. (**c**) Extracted barrier heights *φ*_B_ and ideality factors *n*. (**d**) Forward bias *I*–*V* for samples A and D.

A series of Pt/Nb:SrTiO_3_ devices (Fig. 1a) were investigated, as summarized in Table 1. The interface quality was varied intentionally across this series. The samples are labelled A–D, in the order of decreasing interface quality. Sample A (highest quality interface) consists of an all-epitaxial junction of (001)Pt grown by high-temperature sputtering^13^. The interface quality of samples B, C and D was progressively reduced by sputtering Pt at room temperature (B), removing the *in situ* pregrowth anneal (C) and using highly energetic deposition (D). All samples were annealed in O_2_ to eliminate any possible contributions from oxygen vacancies.

Figure 1b shows the *I*–*V* characteristics of the four Pt/Nb:SrTiO_3_ junctions. The *I*–*V* hysteresis was probed by sweeping from −6 V to +2 V, then back to −6 V. All samples show bipolar resistive switching, with a high positive bias increasing the junction current and negative bias reversing the effect. The device states are referred to as low- and high-resistance states (LRS and HRS). The HRS is identical to the initial state of the device if an appropriate switching protocol is chosen. The conductivity of the LRS can be tuned by modifying this protocol^7^. The magnitude of the resistive switching clearly decreases as the junction quality is improved (that is, from sample D to A).

At low forward bias, the junction current can be described by thermionic emission theory:


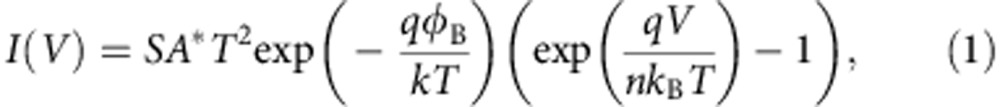


where *S* is the junction area, A^*^ the Richardson constant, *q* the electron charge, *k* the Boltzmann constant, *T* the temperature, *n* the ideality factor and *φ*_B_ the Schottky barrier height. The ideality factor *n* describes the deviation from ideal thermionic emission:


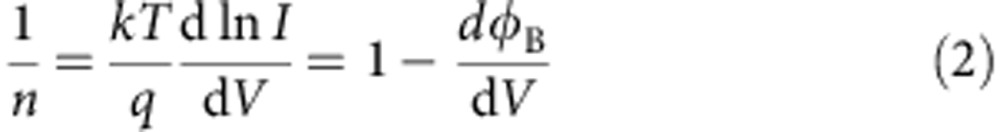


Here, we use Equation (1) with *φ*_B_ and *n* as fit parameters to describe the lower and upper branches of the forward bias loop, corresponding to the HRS and LRS, respectively. The results are shown in Fig. 1c, from which several systematic trends are evident. Specifically, higher quality junctions have lower *n* in both HRS and LRS and slightly lower *φ*_B_ in HRS. Switching to LRS increases *n* and reduces *φ*_B_, and this effect is much more pronounced in lower quality junctions.

The value of *n*=1.19 for the junction with epitaxial Pt (sample A) in LRS is very close to the ideal value (*n*=1). The near-ideal value is particularly noteworthy considering the high-doping level in the SrTiO_3_ (*N*_*D*_=10^20^ cm^−3^), because *n* typically increases with *N*_D_^8^,^14^,^15^. The lowest reported value at room temperature is *n*=1.14^16^ for an *in situ* ozone-cleaned Au/Nb:SrTiO_3_ junction, albeit at a much lower doping of *N*_D_=10^18^ cm^−3^. At high-doping levels (*N*_D_>10^18^ cm^−3^), typically reported values of *n* are above 1.4^8^,^9^, similar to our samples B, C and D.

Two possible origins exist for *n*>1: (i) electron tunnelling through the barrier and (ii) a voltage-dependent barrier height^8^,^14^. In case of (i), the forward bias current should increase with *n*. Figure 1d shows that the opposite is true: the forward current is higher at low *n*. This suggests mechanism (ii), which is generally linked to the presence of an insulating interface layer and/or surface states^15^,^17^, the combination of which causes *n*>1. Next, we further analyse *n*, which allows for insights into the origins of resistive switching.

The applied bias *V* is partitioned by the parasitic capacitance of the interface layer *C*_i_, with *V*_i_=*V*(*n*−1)/*n* applied across the interface layer. The voltage across the depletion region is thus reduced to *V*_D_=*V*/*n*. A complete description of *n*^15^,^17^ involves not just *C*_i_, but also the surface state charge, separated into two parts: the charge density in equilibrium with the metal (*D*_sa_) and with the doped SrTiO_3_ (*D*_sb_):


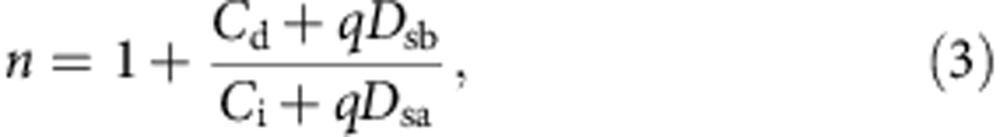


where *C*_d_ is the depletion region capacitance. An adequate description of metal/Nb:SrTiO_3_ junctions can usually be obtained by neglecting the interface trapped charge^9^,^10^,^14^, which does not play a dominant role in *C*–*V* data measured at high frequencies (discussed next). In this case, *n* is given as:


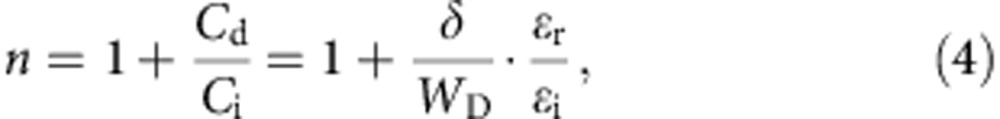


where *δ* and *W*_D_ are the interface layer and depletion width thicknesses, and *ε*_i_ and *ε*_r_ are their respective dielectric constants. From Equation (4), we see that the increase of *n* with decreasing interface quality can be rationalized in terms of a smaller interface capacitance caused by an increased thickness of the interfacial layer (*δ*). The increase in *n* and the lowering of *φ*_B_ on switching the junction to the LRS are generally observed for this type of resistive switching memory. It has been ascribed to barrier inhomogeneity^8^,^11^,^18^,^19^,^20^, because the global *φ*_B_ as measured in photocurrent experiments is insensitive to resistive switching^19^,^21^.

### Capacitance–voltage characteristics

**Figure 2:**
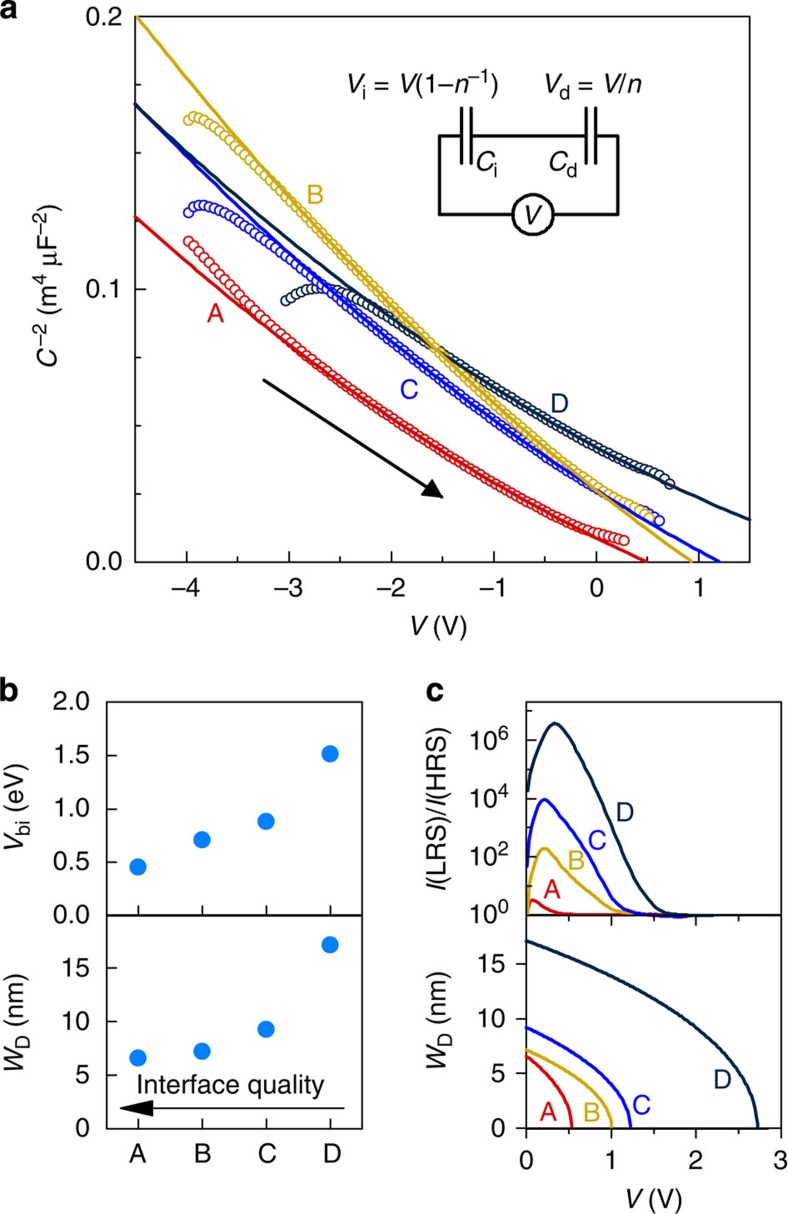
*C*–*V* characteristics of the devices. (**a**) *C*–*V* data for all samples in the HRS, plotted as *C*^−2^ versus *V*. The lines are fits to Equation (6). The inset shows the equivalent circuit model and the voltage partitioning between the depletion and interface layer capacitances, *C*_d_ and *C*_i_. (**b**) Extracted built-in potentials *V*_bi_ and depletion widths *W*_D_ at zero bias. (**c**) Ratio between the currents in LRS and HRS as the function of voltage (top) and the calculated depletion width under forward bias (bottom).

Further evidence of the interface layer is provided by capacitance–voltage (*C–V*) measurements. As discussed next, *C–V* clearly show the effects of applied bias partitioning between the depletion width and the interface layer. Figure 2a shows *C*^−2^ as a function of bias for all junctions (HRS), measured at 1 MHz frequency. On switching to LRS (not shown), the capacitance is hardly affected, in contrast to the large effect on *I*–*V* characteristics. The capacitance in LRS is increased by only a few per cent and decays with time, similar to the trend observed in the *I*–*V* measurements^6^,^11^.

For Schottky junctions with a conventional semiconductor, *C*^−2^ is linearly dependent on *V*, as given by *dC*^−2^/*dV=−*2/(*eε*_0_*ε*_1_*N*_D_), where *ε*_0_ and *ε*_r_ are the vacuum dielectric permittivity and semiconductor relative dielectric permittivity. SrTiO_3_ has an electric field (*E*) dependent permittivity, which can be described as:


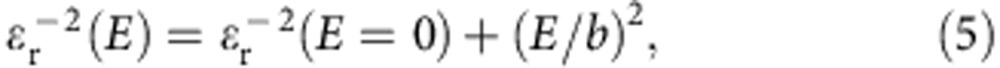


where *ε*_r_(*E=*0) and *b* are temperature-dependent constants. The field-dependent permittivity is responsible for the curvature of the *C*^−2^*–V* curves seen in Fig. 2a. The reduced curvature of the junctions with high *n* can thus be explained by the presence of an interface layer, which reduces the electric field that drops over the depletion region. Quantitatively, the measured capacitance is *C*=*C*_d_/*n*, and for a field-tunable *ε*_r_ it can be written as^14^,^17^:


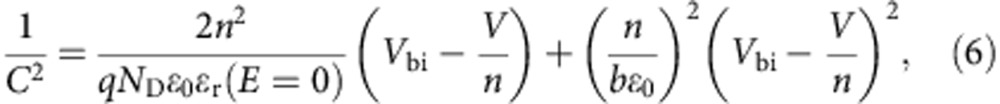


where *V*_bi_ is the built-in voltage of the junction. We use this expression to fit the data in Fig. 2a, using *V*_bi_ and *N*_D_ as the fit parameters. The constants *ε*_r_(*E=*0) and *b* were extracted from the voltage dependence of *C* as described in refs 14, 22 and in the Methods Section. Using *n* obtained in the HRS from the *I–V* curves, the extracted values for *N*_D_ are within 4% of *N*_D_ determined by Hall measurements. The good description provided by Equation (6) confirms the interface layer model and shows that Equation (4) is appropriate. In particular, the analysis shows that *n* increases with decreasing interface quality because of the decrease of *C*_i_ in Equation (4), and not because of the interface states (see Equation (3)). Specifically, *δ* determines the *C*_i_ and the magnitude of *n*, and results in partitioning of the applied bias between the depletion width and the interface layer. We note that we make no claims that the interface states are absent (see also below), only that they are not the cause for the observed trend of increasing *n* in low-quality junctions.

Figure 2b shows that the extracted *V*_bi_ decreases with junction quality, similar to the trend observed for *φ*_B_. The magnitude of the resistive switching clearly scales with *V*_bi_, similar to prior reports as a function of electrode metal work function^9^. *V*=*nV*_bi_ represents the forward bias at which the depletion region vanishes. The depletion width, *W*_D_, can be calculated from *V*_bi_^14^,^17^:


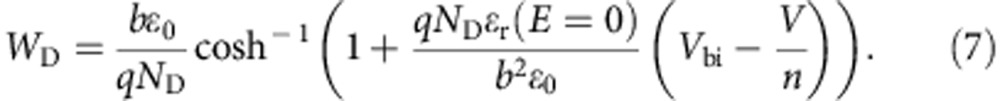


The zero-bias value of *W*_D_ is shown in Fig. 2b for all junctions. It scales with junction quality, similar to *V*_bi_. Figure 2c (bottom graph) shows the calculated *W*_D_ as a function of forward bias. The difference between *I*(LRS) and *I*(HRS) goes through a maximum with applied voltage, and the hysteresis loop closes (*I*(LRS)=*I*(HRS)) approximately at the same voltage when *W*_D_ vanishes (see top graph in Fig. 2c).

### Switching between resistance states

**Figure 3:**
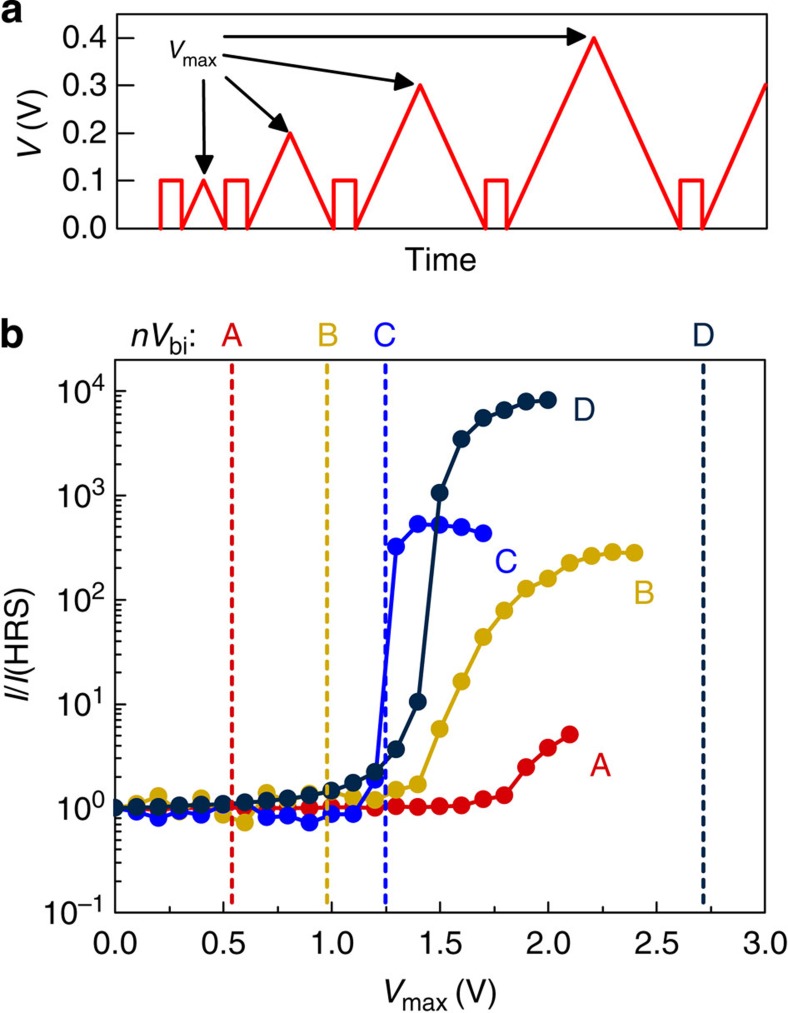
Switching characteristics of the devices. (**a**) Applied voltage sequence used for testing the switching from HRS to LRS. The sequence consisted of alternating between 0.1 V read pulses and increasingly large switching biases. (**b**) Normalized small-signal currents plotted as a function of peak voltage in the switching loop. The effective built-in voltages, *V*=*nV*_bi_, are indicated by the dashed lines.

It is important to note that the switching from HRS to LRS occurs at a higher forward bias than that required for *W*_D_=0. This is illustrated in Fig. 3, where a sequence of positive bias loops with increasing peak voltages (*V*_max_) was applied (top graph), measuring the low-signal current (at +100 mV, bottom graph) between each cycle. As *V*_max_ is increased, switching from HRS to LRS is observed for all devices. Except for junction D (lowest quality), the threshold for resistive switching is clearly above *V*=*nV*_bi_ (dashed lines in Fig. 3c). The threshold voltage for switching is larger for high-quality junctions, showing the opposite trend as *V*_bi_. As there is no more depletion region when the junction transitions from HRS to LRS, it is unlikely that defects that are located within the depletion region at zero bias are responsible for large resistive switching.

## Discussion

**Figure 4:**
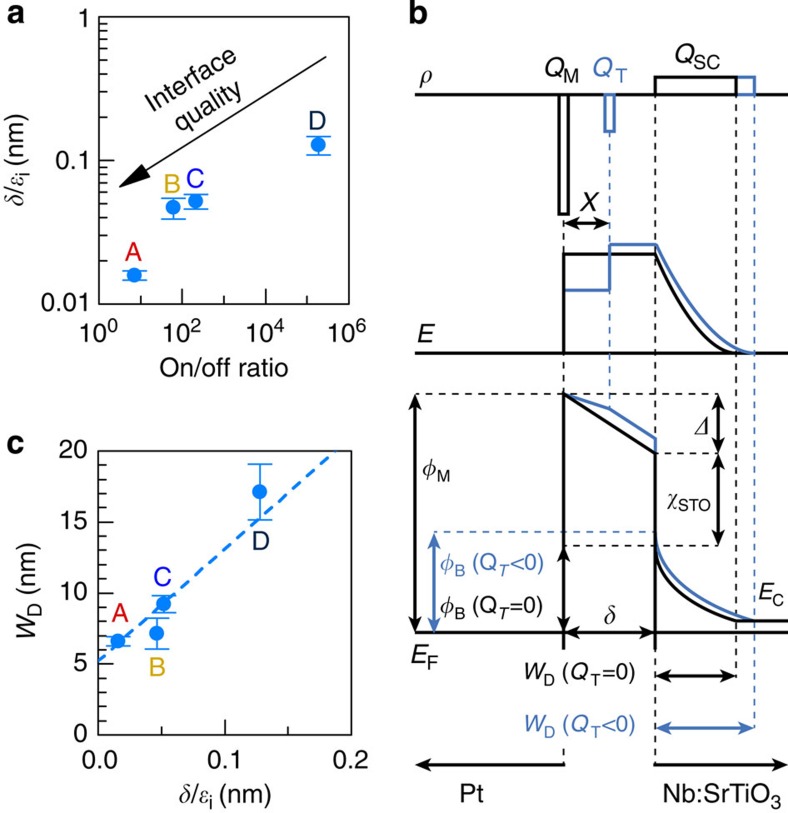
Influence of the interface layer on the Schottky junction parameters. (**a**) Correlation between the magnitude of resistive switching and the interface layer thickness. (**b**) Effect of a negative trapped charge *Q*_T_ on a Pt/Nb:SrTiO_3_ junction. Top: charge density (*ρ*) distribution profile with and without *Q*_T_. Middle: electric field (*E*) profile with and without *Q*_T_. Bottom: band profile. The Schottky barrier is increased by a negative *Q*_T_. (**c**) Correlation between the zero-bias depletion width and the interface layer thickness.

To briefly recap, the main result so far is the trend shown in Fig. 1a, namely the progressive suppression of resistive switching in high-quality junctions. From the combined analysis of *I*–*V* and *C*-*V* data, this trend can be explained by the reduction of an interface layer thickness *δ*. This correlation is illustrated in Fig. 4a, where the on/off ratio (*I*(LRS):*I*(HRS) measured at 0.1 V immediately after switching to LRS and HRS), is plotted against *δ*/*ε*_*i*_, calculated from Equation (4). For interfaces with near-ideal *n*, resistive switching and the interface layer are (nearly) absent.

Possible physical origins of the interface layer are: (i) unintentional contamination, (ii) growth-induced damage and/or disorder and (iii) an intrinsic deadlayer arising from surface reconstructions^23^. Regarding (i), it is known that oxide surfaces such as SrTiO_3_ chemisorb carbon-hydroxyl layers upon air exposure and removal requires high temperatures and/or oxygen containing atmospheres^24^,^25^,^26^, as applied to sample A. Secondary ion mass spectrometry (SIMS; see Methods section) indicates significant amounts of carbon at the Pt/Nb:SrTiO_3_ interface for samples B, C and D, consistent with an interface contamination layer. (ii) may be an additional factor for sample D, as e-beam evaporation is known to cause more damage from energetic deposition than sputtering^27^. Intrinsic deadlayers, (iii), are unlikely to play a significant role, because of the systematic trend with interface processing. Oxygen vacancy concentrations were minimized by post-growth annealing in oxygen and, even if present, are the same for all samples. Consequently, a mobile defect migration-based resistive switching mechanism is highly unlikely to be relevant for resistive switching in the material system studied here (though it may, of course, play a role in other types of resistive switching memory devices).

The most likely mechanism for resistive switching involves charge trapping within the interface layer, as will be discussed next. Figure 4b shows the electric field profile across a junction that contains an effective trapped charge *Q*_T_ with a centroid position *x*, which could be within the interface layer or at the interface or both. *Q*_T_ alters the electric field profile across the junction. For example, a negative *Q*_T_ results in increased *φ*_B_ and *W*_D_. The effect of *Q*_T_ is accounted by an energy Δ, which modifies *φ*_B_ from its ideal value^28^, as described by:


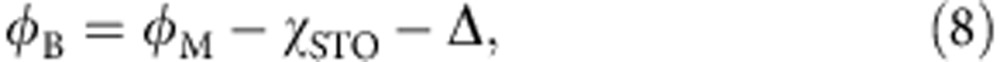


where *φ*_M_ is the metal work function and *χ*_STO_ is the electron affinity of SrTiO_3_. Both *Q*_T_ and the space charge *Q*_SC_***=**qN*_D_*W*_D_ in the depletion layer contribute to Δ. For a charge centroid at *x* (see Fig. 4b):


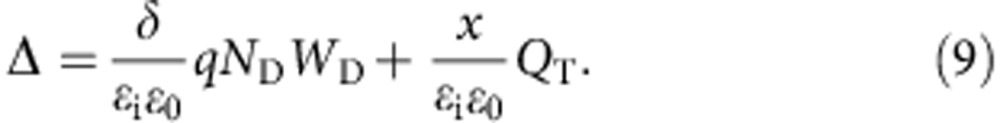


The value of *φ*_B_ is thus dependent on the spatial distribution of the trapped charge. Combining Equations (8) and (9), we obtain:


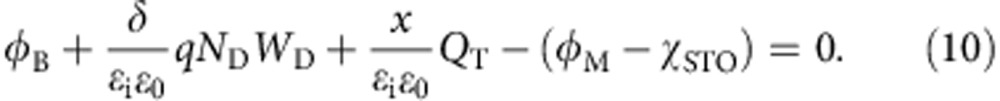


**Figure 5:**
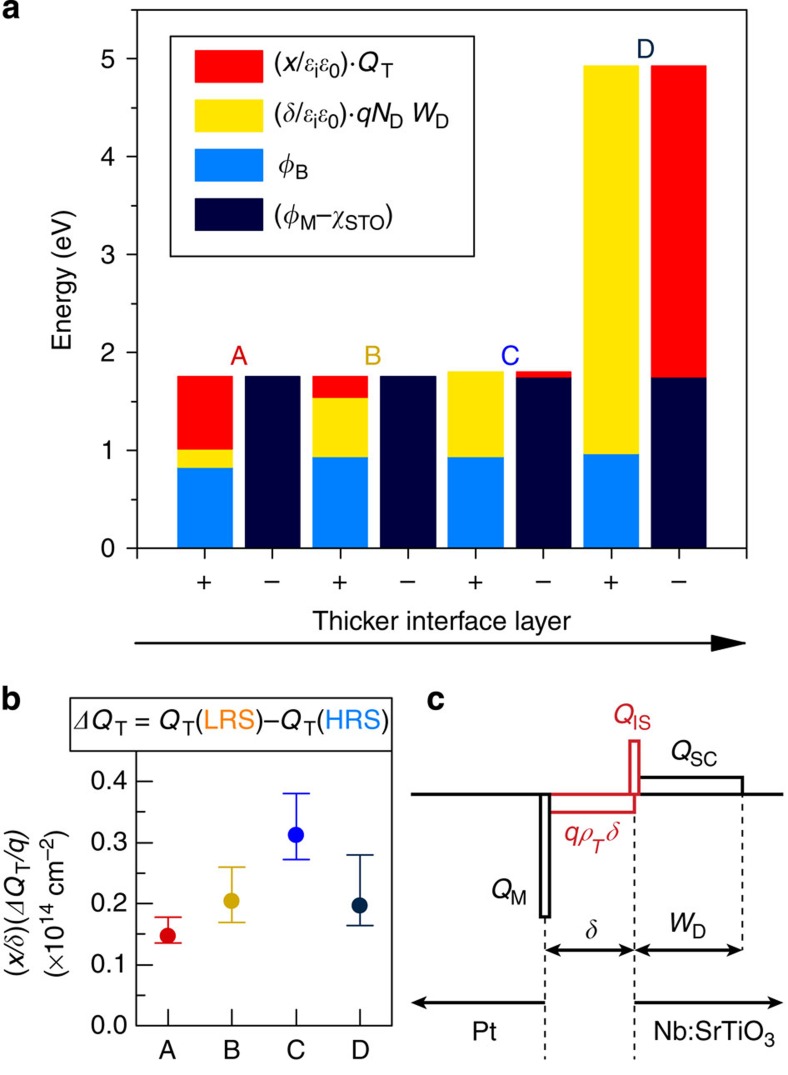
Contributions of trapped and interface charges. (**a**) Magnitude of the positive and negative terms (+ and − columns, respectively) in Equation (10) for all junctions in HRS. (**b**) Calculated modulation of trapped charged centroid during resistive switching, Δ*Q*_T_=*Q*_T_(LRS*)−Q*_T_(HRS). (**c**) Simplified charge density profile for a Pt/Nb:SrTiO_3_ junction with interface states and trapped charge in the interface layer.

The first two terms in Equation (10) are known from the *I*–*V* and *C*–*V* measurements. The last term is constant across the series, and we take *φ*_M_=5.65 eV^29^ and *χ*_STO_=3.9 eV^30^. The third term, which contains *Q*_T_, can thus be obtained from the experimental data. Figure 5a illustrates the contributions of all four terms in Equation (10) for the four samples. They are grouped into the negative [(*φ*_M_−*χ*_STO_)=1.75 eV] and positive (*φ*_B_ and 
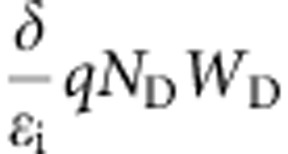
) terms in Equation (10). *Q*_T_ can of course be negative or positive, and furthermore, it may contain contributions from interface traps and trapped charge within the interface layer. From Fig. 5a, we see an increasingly large contribution from the depletion layer for the samples with high *δ*. Because (*φ*_M_−*χ*_STO_) is constant, Equation (10) yields a contribution from *Q*_T_ that switches from positive to negative sign across the series. The crossover from positive to negative can be rationalized by the presence of two distinct contributions to *Q*_T_, with opposite signs. One contribution must come from the interface states (*Q*_IS_) at the interface with SrTiO_3_. In general, *Q*_IS_ is determined by defects that are intrinsic to a specific semiconductor’s surface and is relatively insensitive to the specific overlayer^31^,^32^. Depending on the position with respect to the charge neutrality level (CNL) results, *Q*_IS_ can be negative or positive^28^. In SrTiO_3_, the CNL is high in the upper half of the band gap, approximately 0.7 eV below the conduction band^30^,^33^. Given the measured *φ*_B_ heights in Fig. 1c and the degenerate nature of doped SrTiO_3_^34^, this implies that the Fermi level is below CNL and *Q*_IS_ is positive. This suggests a second contribution to *Q*_T_, which is negative, and increases with decreasing interface quality. Figure 5a shows that it scales with *δ*, which implies a negative trapped charge, *qδρ*_T_, with a volume density *ρ*_T_ in the interface layer. The negative trapped charge also explains the linear scaling of depletion width with *δ.* As shown in Fig. 4c, in the presence of a large negative charge in the interface layer, *W*_D_ increases to satisfy charge neutrality.

The charge profile resulting from the two contributions to *Q*_T_ is illustrated in Fig. 5c. The positive *Q*_IS_ and negative *ρ*_T_ produce the effective charge centroid *Q*_T_, plotted in Fig. 5a,b:


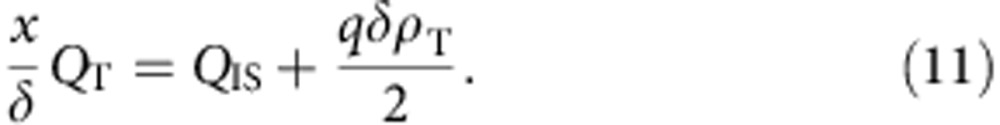


Figure 5c shows the simplest charge profile that can be used to rationalize the scaling of *Q*_T_ with *δ*. Although more complex, inhomogeneous distributions of trapped charge in the interface layer are possible^35^, their role in modifying *φ*_B_ is still correctly described by a charge centroid *Q*_T_.

As shown in Fig. 5b, the analysis also lends itself to an explanation of the switching between HRS and LRS. Switching is accomplished by a change in *Q*_T_ towards a more positive value in LRS, which can happen in two ways: (i) trapping of additional positive charge at the SrTiO_3_ surface, increasing *Q*_IS_, or, (ii) reduction of the negative charge in the interface layer, decreasing *δρ*_T_. Mechanism (ii) is more likely, given the nature of the time-dependent decay of LRS towards HRS.

**Figure 6:**
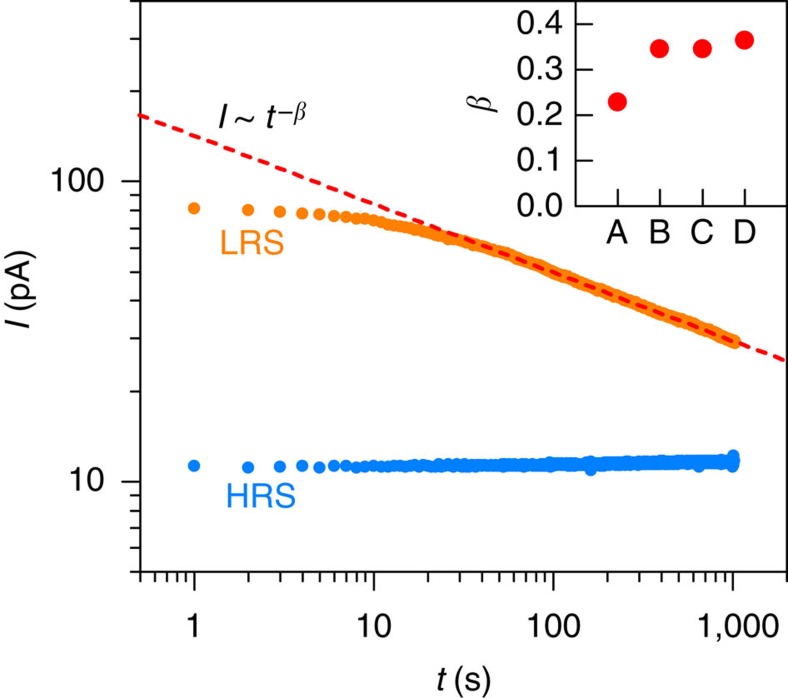
Resistance state retention characteristics. Time-dependence of the small-signal (+0.1 V) current after switching to HRS and LRS, shown for sample A. The red dashed line is a fit to a power law for the decay of LRS. The inset shows the power law exponent for all junctions.

The state retention characteristics of the junctions are shown in Fig. 6. After a positive or negative voltage loop (for HRS and LRS respectively), the small-signal current is monitored (at *V*=+100 mV). With the appropriate switching protocol, the HRS is a stable state, whereas the current in LRS decays with time, eventually returning back to HRS. The decay of the LRS follows a power law with time after switching (*I*~*t*^*β*^) after the junction is switched to LRS^11^,^36^. This so-called Curie-von-Schweidler behaviour is typical for capacitive charging^37^ and it is commonly observed for charge trapping under bias in high-k dielectrics^38^,^39^. The decay is due to a progressive increase in negative charges, which were previously de-trapped upon switching to LRS, implying mechanism (ii). The decay rates are similar for all junctions, with a slight trend towards slower decay in high-quality junctions, as revealed by the exponent *β* (see the inset in Fig. 6). The similarity in the retention properties of all junctions suggests a common origin of their resistive switching properties.

In contrast to the large resistive switching effect in the *I*–*V* measurement, Δ*Q*_T_, the change of *Q*_T_ between HRS and LRS (Fig. 5b), does not correlate with *δ*. Instead, it scales with the threshold voltage for resistive switching, shown in Fig. 3. The variation of Δ*Q*_T_ across the series is quite small. This implies rather similar defect chemistry across the series that drives the resistive switching, and it further emphasizes the crucial roles of the interface layer thickness (capacitance): The Schottky barrier modulation (Fig. 1c) is caused by ΔQ_T_, but the magnitude of the effect is determined by interface layer capacitance (thickness), as is evident from Equations (9) and (10). The interfacial layer capacitance (ε_i_/δ) determines the voltage drop due to the trapped charge, thus controlling the degree of Schottky barrier modulation, and the magnitude of the resistive switching effect, upon the voltage-induced modulation of the trapped charge. Consequently, the magnitude of the resistive switching effect directly scales with *δ*, as seen experimentally in Figs 1b and 4a. Fluctuations in interface capacitance between devices, caused by variations in *δ*, can readily explain the commonly observed non-uniformity of resistive switching parameters.

In summary, the results presented here establish a strong connection between the suppression of resistive switching and reduction of an interfacial layer. To date, research on this type of device has largely focused on Schottky contacts using metals deposited at room temperature. As shown here, such interfaces readily provide large resistive switching effects. The effect is, however, due to an unintentional defective layer that is difficult to control, and thus likely responsible for poor reproducibility and device-to-device variations. The fact that this is now understood, and that it can furthermore be suppressed, using appropriate fabrication methods, opens the way towards engineering resistive switching based on intentional modification of interfaces or defect densities. To produce reliable and reproducible results, and thus a practical technology, high-quality interfaces, such as the epitaxial Pt junctions developed here, are essential to minimize contributions from unintentional layers.

## Methods

### Device processing

Devices were fabricated using (001) SrTiO_3_ single crystals doped *n*-type with 0.7 wt % Nb. To obtain a flat, stepped surface, the substrates were etched in Aqua Regia and annealed at 1,000 °C for 2 h in air. For sample A, which had the highest interface quality, the substrate was annealed at 825 °C in 10 mTorr O_2_, *in situ*, before growth of epitaxial, (001) Pt by DC sputtering at 825 °C in 10 mTorr Ar using a sputter power of 30 W. A detailed characterization such Pt films has been published elsewhere^13^. X-ray data is shown in Supplementary Fig. 1. For sample B, the substrate was cooled after the *in situ* anneal and Pt growth was performed at room temperature. For sample C, the pregrowth anneal was omitted. For sample D, Pt was deposited by standard e-beam evaporation at room temperature. Compared to sputtering, e-beam evaporation produces more damage from energetic deposition^27^. All samples were post-Pt deposition annealed at 800 °C in flowing O_2_ to eliminate any possible contributions from oxygen vacancies that may have formed during Pt deposition. Square 30 × 30 μm^2^ Pt electrodes were patterned by standard photolithography, following by selective oxidation of the top Pt surface by a room temperature plasma in 300 mTorr O_2_ at 100 W. The Pt oxide was then used as a hard mask for wet etching Pt in Aqua Regia at 60 °C^40^. A ground-signal-ground electrode configuration was then completed with Ohmic Al contacts made to the Nb:SrTiO_3_, which was defined by a standard lift-off process (see Fig. 1a for the completed device). *I*–*V* measurements were performed using a needle probe station and a HP 4155 semiconductor parameter analyser. *C*–*V* measurements were performed using a Cascade Microtech probe station with GGB 100-μm ground-signal-ground probes and a HP 4294 impedance analyser at 1 MHz.

### Fitting procedures

Fitting in Fig. 1b was done in the data range below 1 μA to minimize the effect of series resistance. Fitting in Fig. 2a was performed in the range between −2 V and −0.25 V, where the AC conductance was low for all junctions. The s.d. of the extracted fit parameters were <2% for *n* and <1% for *φ*_B_ and *V*_bi_, error bars in Figs 4 and 5 were calculated using error propagation.

To account for the field dependence of the dielectric constant, *ε*_r_, of Nb:SrTiO_3_, we follow the procedure described in refs 14, 22. For a metal/semiconductor Schottky junction, the electric field decreases with the distance *z* from the interface. For a non-linear dielectric, this results in a spatial variation of *ε*_r_, which is reduced at the interface (*z*=0) and increases to its zero-field value across the depletion width. The voltage dependence of capacitance can be used to extract the electric field *E* and *ε*_r_ at *z*=0:


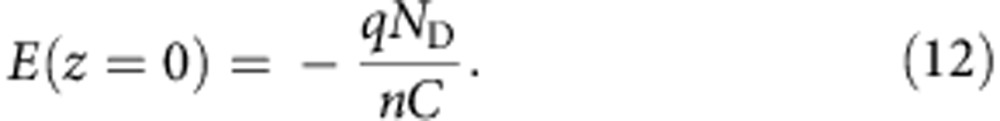



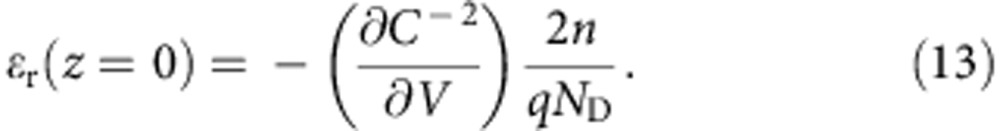


The field dependence of the tunable dielectric constant *ε*_r_ can be parameterized as:


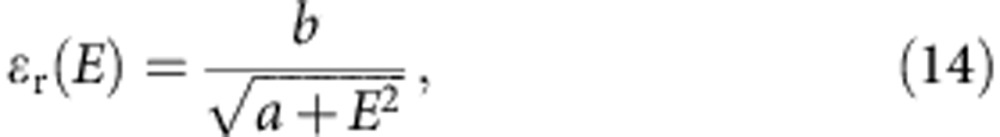


where *a* and *b* are materials constants. The zero-field dielectric constant is then given as: 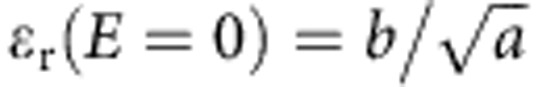, and one can rewrite Equation (14) as a linear relationship between 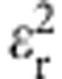 and *E*^2^:


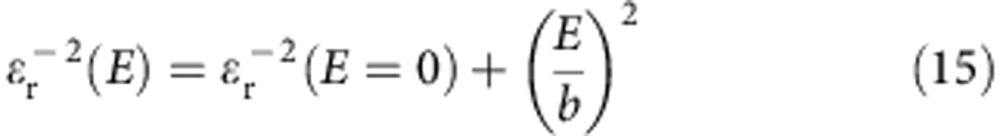


Supplementary Fig. 2 shows 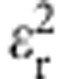 as a function of *E*^2^ at *z*=0, obtained from the experimental *C*–*V* measurements. Fitting to equation (15) in the low-conductance region was used to obtain the materials parameters *b* and *ε*_r_(*E*=0). We also performed fits using a simplified expression, which neglects the field dependence of *ε*_r_, but does account for the voltage partitioning:


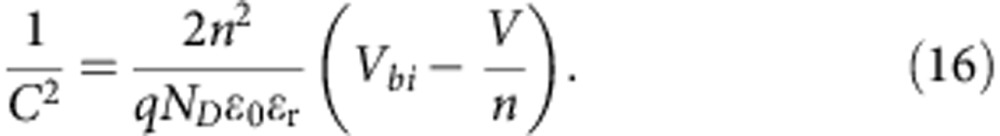


The fit parameters here are *ε*_r_ and *V*_bi_. The values of ideality factors *n* are taken from HRS in the *I*–*V* measurement. As shown in Supplementary Fig. 3, this linear model provides a good description only in the low negative voltage region. However, the extracted values and trends for *ε*_r_ and *V*_bi_ are fairly close to the ones deduced from the non-linear model. Consequently, the *ε*_r_ obtained with the linear model is a good approximation for the integrated effect of the depletion width capacitance, although in reality it has a non-uniform profile of *ε*_r_^14^. These *ε*_r_ values are used in the main text for the purpose of quantifying the effect of trapped charges. Supplementary Table I summarizes the dielectric properties extracted from the fits of *C*–*V* data for all junctions, using both models outlined above. Supplementary Table II summarizes the Schottky barrier and state retention properties extracted from *I*–*V* measurements.

### SIMS

**Figure 7:**
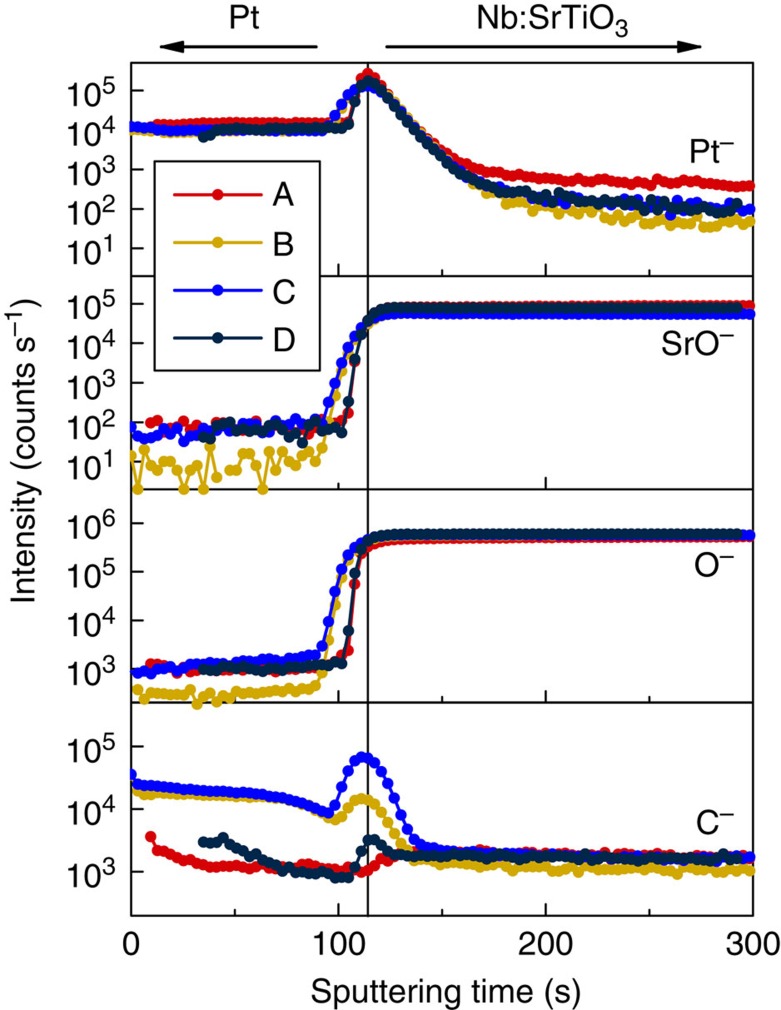
Interface chemistry of all four samples. Shown are the SIMS depth profiles of Pt^−^, SrO^−^, O^−^ and C^−^ for all samples. For samples B, C and D, a carbon peak is detected at the Pt/Nb:SrTiO_3_ interface.

SIMS was performed using a Physical Electronics 6650 Quadrupole instrument (Physical Electronics, Chanhassen, MN). A 6 kV, 100 nA caesium primary ion beam with a spot size of 60 μm was rastered over a 300-μm-diameter area, and secondary ions were accepted from the centre 15 per cent of the rastered areas. A low-voltage electron beam was used for charge neutralization. Fig. 7 shows SIMS Pt, Sr, O and C profiles for all junctions. Sr and O show a sharp transition at the SrTiO_3_/Pt interface. Pt shows a rise in intensity at the interface, most likely associated with increased ionization yield in presence of oxygen sputtered from SrTiO_3_ at the interface. The magnitude of this rise is uniform across the studied sample series. The carbon profiles in samples B, C and D show increased intensity at the interface due to an unintentional interface contamination layers. It is unrelated to the increased Pt ionization yield near the interface. The absence of the carbon peak at the interface of sample A is consistent with the high quality of the interface, which was grown at 825 °C, which is likely sufficient to remove contamination layers.

## Author contributions

E.M., B.D.H. and S.S. conceived and designed the experiments. E.M. and B.D.H. developed the device processing. E.M carried out the experiments and analysed the data. All authors discussed the results.

## Additional information

**How to cite this article**: Mikheev, E. *et al.* Resistive switching and its suppression in Pt/Nb:SrTiO_3_ junctions. *Nat. Commun.* 5:3990 doi: 10.1038/ncomms4990 (2014).

## Supplementary Material

Supplementary Information Supplementary Figures 1-3 and Supplementary Tables 1-2

## Figures and Tables

**Table 1:** Summary of the Pt/Nb:SrTiO_3_ junction fabrication

**Sample**	**A**	**B**	**C**	**D**
Pt deposition technique	DC sputtering	DC sputtering	DC sputtering	E-beam evaporation
*In situ* pregrowth anneal	2 h, 825 °C	2 h, 825 °C	5 min, 120 °C	None
Pt growth temperature	825 °C	Room temperature	Room temperature	Room temperature
Pt microstructure	Epitaxial, (001)-oriented	Polycrystalline	Polycrystalline	Polycrystalline
Post-growth anneal	30 s, 800 °C in flowing O_2_	30 s, 800 °C in flowing O_2_	30 s, 800 °C in flowing O_2_	30 s, 800 °C in flowing O_2_
