# Correction: Corrigendum: Resistive switching and its suppression in Pt/Nb:SrTiO_3_ junctions

**DOI:** 10.1038/ncomms10128

**Published:** 2015-12-07

**Authors:** Evgeny Mikheev, Brian D. Hoskins, Dmitri B. Strukov, Susanne Stemmer

10.1038/ncomms4990

In Fig. 2a of the main manuscript and in Supplementary Fig. 3, the unit label for *C*^−2^ (on the vertical axis) should read (cm^4^/μF^2^) instead of (m^4^/μF^2^).

This correction only applies to the labels in these two figures and does not affect any of the calculations or conclusions in the paper.

