# Supplementary material for: Resistive switching and its suppression in Pt/Nb:SrTiO3 junctions
Source: Nat Commun. 2014 Jun 2;5:3990. doi: 10.1038/ncomms4990 (PMC4059928; doi:10.1038/ncomms4990)
Supplement: Supplementary Information — Supplementary Figures 1-3 and Supplementary Tables 1-2 [file ncomms4990-s1.pdf]

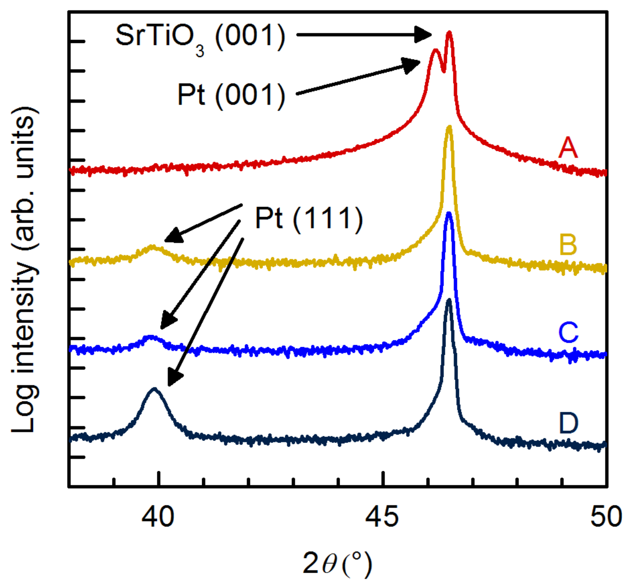

Supplementary Figure 1: X-ray diffraction 2θ-ω scans of all samples. Sample A, grown at 825 °C, reveals a single strong 001_{Pt} peak, confirming epitaxial growth. The polycrystalline samples B, C and D show 111_{Pt} peaks.

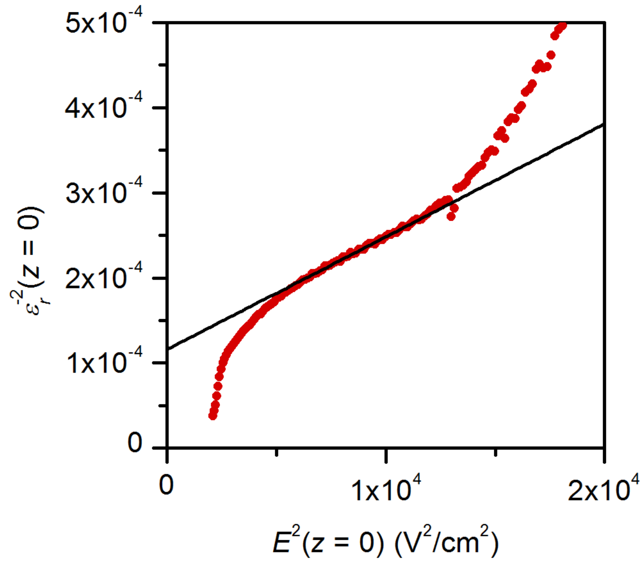

Supplementary Figure 2: ε_r^{2} - E^2 relationship at $z = 0$ (Pt/Nb:$SrTiO_3$ interface) for sample A, obtained from the *C*-*V* measurements shown in Fig. 2a, and using equations (12) and (13). The black line is a fit to Eq. (15).

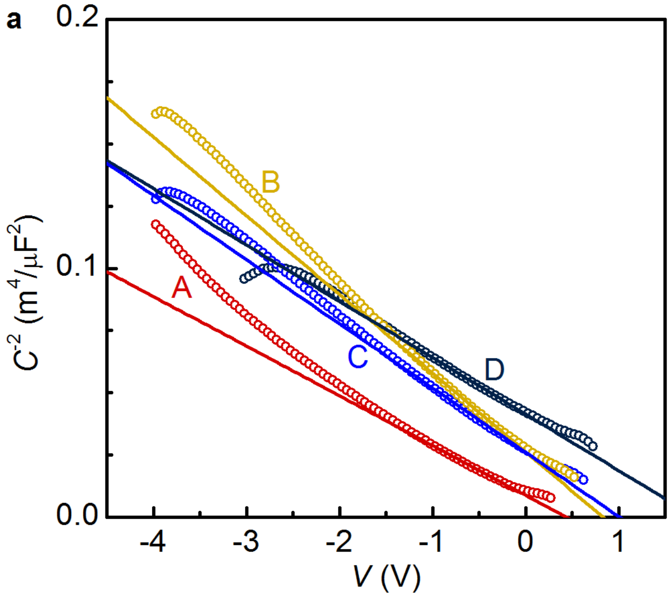

Supplementary Figure 3: Capacitance-voltage data for all samples in the HRS, plotted as C^{-2} vs. V. The lines are fits to a simplified model assuming a non-field dependent dielectric constant in $SrTiO_3$ [Eq. (16)].

Supplementary Table I: Summary of dielectric properties extracted from *C-V* measurements.

Sample	*C-V* (HRS)				
	Non-linear ε_r model			Linear ε_r model	
	b (V/cm)	ε_r ($E = 0$)	V_{bi} (eV)	ε_r	V_{bi} (eV)
A	2.75×10^5	92.9	0.45	80.4	0.40
B	2.77×10^5	70.3	0.71	59.0	0.63
C	2.54×10^5	94.4	0.88	73.76	0.74
D	2.75×10^5	206.4	1.51	106.6	1.04

Supplementary Table II: Summary of Schottky barrier and state retention properties extracted from *I-V* measurements.

Sample	*I-V* (HRS)		*I-V* (LRS)		State retention	
	n	ϕ_B (eV)	n	ϕ_B (eV)	ON/OFF ratio at $t = 0$ s	β
A	1.19	0.83	1.39	0.79	$1.8 \cdot 10^5$	0.23
B	1.39	0.94	1.84	0.77	220	0.35
C	1.42	0.94	2.07	0.64	62	0.34
D	1.80	0.97	2.74	0.51	7.1	0.36
